# Researching religion and migration 20 years after ‘9/11’: Taking stock and looking ahead

**DOI:** 10.1007/s41682-022-00103-6

**Published:** 2022-03-02

**Authors:** Fenella Fleischmann

**Affiliations:** Department of Sociology, University of Amsterdam, Nieuwe Achtergracht 166, 1018 WV Amsterdam, The Netherlands

**Keywords:** Religion, Migration, Integration, Religiosity, Religious attitudes, Religious cognition

## Abstract

This contribution to the special issue on religion and migration reviews two decades of large-scale survey research on changes in immigrant religion and the relationship between immigrants’ level of religiosity and their integration into European societies. The body of work reveals that Muslims in European societies stand out due to their comparatively high levels of religiosity and greater stability in religiosity over time and across immigrant generations. While the comparative picture is rather clear, findings regarding the long-term trend in Muslims’ religiosity and its association with immigrant integration are instead inconclusive. A systematic review of empirical studies of the association of (various indicators of) individual religiosity with immigrant integration reveals positive, negative and non-significant results for all outcomes and domains. Thus, based on the current state of art it is hard to assess whether and why religion forms a bridge or barrier to immigrant integration in Europe. To move the field forward, the contribution ends with a twofold proposal for a research agenda that includes a broadened empirical scope, moving beyond the focus on Sunni Muslims, and a conceptual extension that focuses on differences in reasoning about religion and religious meaning-making as additional, potentially more consistent and more powerful explanation for immigrants’ social relations and positions in their new societies

## Introduction

While the COVID-19 pandemic continues to claim headlines of newspapers worldwide and climate change moves to the centre of public and policy debates, another topic looms large and regularly reminds us of the other unsolved challenge of our time: international migration, particularly the unregulated form from less developed countries to the WEIRD^1^ nations. Migrants from the Middle East being used as human chess pieces in the geopolitical strategy game between Belarus and the European Union, record numbers of migrants crossing the Channel despite the largest number of casualties after a capsizing, and a continuous stream of overcrowded vessels in the Mediterranean are only some of the recent reminders that migration is a pressing societal issue in need of continued policy attention. Yet the admission of migrants, or even any form of migration governance that is not exclusively directed at keeping migrants outside, meets much resistance in European immigrant receiving societies, as evident in public protests against migration and more particularly the arrival of refugees. In this polarised setting, refugees are routinely equated with or primarily perceived as Muslims (Pickel and Pickel 2019) and this anti-Muslim sentiment, in turn, is a strong driver of voting for right-wing populist parties such as the German AfD (e.g., Pickel and Yendell 2018; Huber and Yendell 2019). Thus, in public and policy debates on migration, the religious dimension of the phenomenon occupies a central position, and this makes the focus of the present special issue on religion and migration timely and urgent.

The social scientific study of religion and migration is situated at the crossroads of multiple disciplinary fields. In the sociology of religion, the migration-induced increase of religious diversity has led to a re-evaluation of theories of religious decline and spiked interest in the effects of religious diversity on the religious affiliation, practices and beliefs of large populations (e.g. Casanova 2009; Koenig and Wolf 2013). As a consequence, this field has shown a growing interest in religious pluralism and the religious expression of immigrants. Similarly, in migration studies, the research interest in migrants’ religiosity and how this relates to their position in their new societies has been increasing since the 1990s. Earlier scholarship in this field was primarily concerned with issues of legal and structural integration (work, housing, citizenship) and cultural characteristics of the new minorities only came to the fore when the notion of temporary labour (‘guestworker’) migration was slowly replaced with the realisation that the presence of newcomers and a steady inflow of new immigrants were no transient phenomena but here to stay.

As a migration scholar, I will not further elaborate on the question of why migration is important to study from the perspective of religious studies. Instead my focus will be on the importance of religion for migration studies, and specifically the question of how religion changes in the context of immigrant integration. I will moreover focus my contribution on the European receiving context, and review studies that have examined religious change and the relation between immigrant religion and integration in the North-Western European societies that have seen the earliest large-scale migration after WWII. Considering that Muslims are the largest religious minority in this context and that societal debates about religious diversity and accommodation have focused on Islam and Muslims in Europe, my contribution will be grounded in research conducted among European Muslims with a migration background.

This contribution is structured as follows. After providing some background on the study of religion in migration research, I will review two decades of empirical research using large-scale data to investigate religious change among immigrants (not limited to, but focusing on Muslims) and the relation between migrants’ religiosity and their integration into historically Christian but increasingly secularised European societies. This body of research addresses two questions: (1) How does religion change in the context of migration? (2) Does religion form a bridge or barrier to immigrant integration? As my review will reveal, findings regarding the overall trend of immigrant religiosity and its association with multiple integration outcomes are rather inconclusive to date. To address these unresolved research questions, the last part of this contribution therefore proposes a research agenda that can potentially reconcile currently inconsistent findings and provide a better answer to the question of how and why immigrants’ religion matters for their participation and social relations in European societies.

## Early research on Islam and Muslims in Europe: focus on institutions

Migration scholars in Europe largely started to investigate immigrant religion from the 1990s onwards (e.g. Gerholm and Lithman 1990). This interest was triggered, on the one hand, by landmark events such as the Rushdie affair in Great Britain and the first *affaire du foulard* in France, which both put a spotlight on the newly established presence of Muslim minorities and sparked heated societal debates about the accommodation of this particular religious minority. At the same time, migrant communities started to invest more into religious infrastructures (e.g. raising funds for purpose-built mosques) after realising that their stay was more permanent than temporary, and particularly after having brought over or started families in their new societies. For immigrant parents who had grown up in a radically different context when it comes to religion, the emergence of a second generation raised a need to invest into organised religious communities in order to safeguard the intergenerational transmission of religion. Acculturation research tells us that religious values and practices often become more salient in families after migration due to the stressors involved in the process, and they figure prominently among the qualities immigrant parents aim to pass on to their children growing up in a different society (Phalet et al. 2018; Suárez-Orozco et al. 2011). In the early stages of researching immigrant religion, many scholars therefore focused on ethnographic examinations of lived religion, particularly among migrant youth and in the family context (e.g. Krieger-Krynicki 1990; Thomä-Venske 1990; Vertovec and Rogers 1998; Yalçin-Heckmann 1994). These studies provided welcome insights into the multiple different ways in which Muslim youth relate to their religious identity and showcased the many daily issues that triggered questions of religious belonging and practice, thus revealing the community need for pastoral advice and community organisation from the bottom-up.

Together with societal debates about religious minority rights and the accommodation of Muslims, the accelerated development of religious infrastructures across migrant-receiving societies led to a significant research focus on religious institutions and legal regulations. Many of the early studies and edited volumes on the religion of Muslim newcomers asked what types of Islamic organisations were present in a given receiving society, what their activities were, how they were positioned vis-à-vis each other and the state in their origin countries, and how they were related to and accommodated by the state in the receiving society (Allievi and Nielsen 2003; Maréchal et al. 2003; Nonneman et al. 1996; Rath et al. 2001; Shadid and Van Koningsveld 2002; Sunier 1996; Vertovec and Peach 1997). These works revealed the internal diversity of Muslim communities in Europe (e.g. between the Turkish state-sponsored Diyanet (Diyanet İşleri Başkanlığı) communities and their then oppositional counterpart Milli Görüş, e.g. Spuler-Stegemann 1998; Sunier and Landman 2015), as a function of distinct migration histories and the different ways in which religion is practiced and organised in migrants’ origin countries. In terms of the accommodation of religious minority rights, we learned about the path-dependency where religious newcomers had to come to terms with existing church-state regimes that were more or less religiously neutral and open towards not previously established religious groups (Bader 2007). For instance, in their comparison of Muslims’ religious accommodation in France, Germany and Britain, Fetzer and Soper (2005) conclude that the British legacy of the Anglican church afforded Muslim minorities more opportunities to safeguard institutional support on the basis of religious freedom as well as the principle of equal treatment, whereas the strict separation between state and church in France closed the door towards greater accommodation, and the German system of privileged state cooperation with selected religious groups (in case: Protestant and Catholic churches and Jewish communities) was slow to reform. Similar studies in other countries revealed the Belgian and Austrian cases to show many commonalities with Germany (Foblets and Overbeeke 2002; Mourao Permoser et al. 2010), whereas the Scandinavian countries rather resemble the British case given their similar histories of state churches (Alwall 2002; Simonsen 2002). The Netherlands stand out as a case due to their history of institutionalised religious pluralism combined with state neutrality. Thus, the Dutch case has been described as offering the farthest reaching institutional support, including state funding for religious schools (Doomernik 1995; Rath et al. 1996).

These differences in the legal position and institutional support of Muslim minorities raised the question whether Muslims would show different patterns of religious change and intergenerational transmission in response to the different opportunity structures that they encountered across European countries. Answering this question called for comparative research on large samples of Muslim minorities across European receiving societies.

## The emergence of large-scale survey research on immigrants’ religiosity

Large-scale survey data that allowed for empirical examinations of levels of religiosity, their development and relation to (specific domains of) immigrant integration became increasingly available from 2000 onwards. On the one hand, surveys specifically targeting immigrant minorities included a growing number of questions about religious identification and practices, in addition to more frequently available measures of religious affiliation and service attendance. On the other hand, general population surveys such as the European Social Survey, which already included a good range of indicators of individual religiosity, allowed for a better identification of foreign-born immigrants and their local-born children as well as a classification of their origin country or region from the 2nd round onwards. After 2010, two important comparative research projects collected longitudinal data on recent immigrants (the SCIP data; Diehl et al. 2016) and immigrant youth (CILS4EU; Kalter et al. 2017) that included measures of religiosity and thus allowed studying within-person religious changes over time. Jointly, these developments in the social scientific research infrastructure induced a move away from a focus on institutions towards the study of the ‘average Muslim’, looking at how their religiosity compares to that of non-Muslim immigrants and non-migrants, how it changes over time and how it relates to their position in European societies. In what follows, I provide an overview of the empirical literature on these questions, divided into two principal research questions: (1) How does the religiosity of immigrants in general, and Muslims in particular, change over time? And (2) How is immigrant religiosity related to multiple dimensions of immigrant integration?

## Changing religion among immigrants

I will first review studies that treat immigrant religion as *explanandum* and ask how immigrants’ religion changes as a result of migration and integration into European receiving societies. An important research question is whether the religiosity of (primarily Muslim) immigrants and their children declines with increasing length of stay as they adapt to more secular host societies. Researchers have addressed this question with four distinct empirical approaches. The first relies on synthetic cohorts and compares the levels of religiosity of the foreign-born first generation with the local-born second generation. The second compares parent-child dyads within immigrant families. The third focuses on the event of migration and compares migrants’ religiosity before and after arrival. The fourth draws on longitudinal data and exploits repeated measures of religiosity to observe within-person changes over time. For most of these approaches, studies focusing exclusively on Muslims as the largest religious minority in Europe, as well as comparative studies that include Christians, Muslims and non-affiliates, have been conducted.

The four approaches put different demands on research data and have unique strengths. The synthetic cohort approach is useful to study large-scale generational changes, but also vulnerable to the potentially differential composition of immigrant groups across generations (e.g. in terms of age and origin country) and the differential selection bias of immigrants from the first vs. the second generation into survey research, particularly if it does not concern migrant-specific data collections (i.e., the least integrated members of migrant communities are least likely to participate). The dyadic approach of parents and children is strong in keeping multiple factors that may affect family members simultaneously constant, but limited due to the inherent age gap in comparing parents and their children. Given well-known life-course effects on religiosity, caution needs to be applied when comparing results from dyadic studies with results from synthetic immigrant generations where age differences are taken into account. Repeated measures of religiosity to study within-person developments are widely considered a ‘gold standard’ in longitudinal research, as idiosyncratic differences between individuals can be taken into account that would otherwise contribute to omitted variable bias in between-person comparisons. However, collecting such data among immigrant populations over a long time period is extremely demanding and costly, which explains why such studies still make up a minority of the empirical evidence base on religious change among immigrants and their offspring.

Methodological differences aside, across these four approaches and European countries, there is a recurrent finding that immigrants in general, and Muslims in particular, display higher levels of religiosity than non-migrants (e.g. Lewis and Kashyap 2013a; Simsek et al. 2018; Van Tubergen and Sindradóttir 2011). However, regarding the long-term trend of religious change, theoretical ideas and findings are much more mixed: is there prevalent decline, pointing towards secularisation, or rather stability, or even religious revival, potentially triggered by reactivity in response to discrimination, social exclusion and a negative public opinion climate?

Studies comparing synthetic immigrant generations often document rather stable levels of religiosity across the first and second generation of Muslim immigrants: this has been found in Britain (Lewis and Kashyap 2013b), France (Soehl 2017), Germany (Diehl and Koenig 2009) and the Netherlands (Beek and Fleischmann 2019). Other studies, however, document declining religiosity in the second generation, both among Dutch Muslims (Maliepaard et al. 2010; Phalet et al. 2008) and immigrants across Europe (Van der Bracht et al. 2013). For Muslims in the Netherlands, where repeated cross-sectional surveys allow over-time comparisons for a longer period, the early finding of intergenerational decline might be due to a period effect as later studies revealed an increase in religiosity, particularly among second-generation Muslims (Maliepaard and Gijsberts 2012; Maliepaard et al. 2012; Huijnk 2018). The CILS4EU data of adolescents in four countries even showed higher levels of religiosity among the second compared to the first generation (Simsek et al. 2018). This first approach thus does not reveal consistent findings, but studies documenting increase seem to be outnumbered by those showing decline with stability between the first and second generation as most prevalent outcome.

When religious change is studied through the lens of parent-child dyads, the prevalent finding is a decline in levels of religiosity such that adolescents and (young) adults of immigrant offspring are found to be less religious than their parents (Simsek et al. 2018; Van de Pol and van Tubergen 2014). The discrepancy with results based on synthetic cohorts might be explained from the age gap that is inherent in comparisons of parents with their children, which unavoidably implies a comparison across different life-stages. There is both qualitative and quantitative evidence attesting to the importance of life-course events for differential levels of religiosity, e.g. youngsters indicating ‘not being ready yet’ for living the life of a good Muslim as expected from their parents and religious communities (e.g. Shirazi and Mishra 2010), the well-known spikes in religious participation around life-course transitions and family events (marriage, childbirth, death, e.g. Ingersoll-Dayton et al. 2002) and the use of religion as a coping strategy among elderly people to increase well-being in the face of physical decline, decreased societal participation and loneliness (e.g. Thauvoye et al. 2018). It is therefore doubtful whether findings of intergenerational decline based on parent-child dyads should be interpreted as the onset of large-scale secularisation among Muslim minorities.

The finding that Muslim minorities are more successful in transmitting their religion to their local-born children than Christian immigrants and natives (e.g. Jacob and Kalter 2013; Scourfield et al. 2012; Simsek et al. 2018) also speaks against this scenario of substantial intergenerational decline in this particular religious minority. Moreover, parental religiosity is the strongest predictor of the religiosity of their children, also in the migration context (Güngör et al. 2011; Maliepaard and Lubbers 2013; Van de Pol and van Tubergen 2014), which suggests that the initially high levels of religiosity of the foreign-born first generation are reproduced to a quite significant extent in later immigrant generations.

When it comes to the effect of the migratory event on the religiosity of migrants in Europe, the retrospective measures of pre-migration religiosity available in SCIP show that migration initially lowers the religiosity of both Christian and Muslim newcomers, and is particularly disruptive for the religious practice of service attendance in a church or mosque (Van Tubergen 2013). When recent immigrants are followed over a longer period after their arrival in Germany, however, this initial decline is subsequently reversed and religious practices increase again (Diehl and Koenig 2013). This suggests that the migration-induced decrease in religious practice is a more transient phenomenon and not the onset of extensive religious decline. Finally, analyses of repeated measures of subjective importance of religion, prayer and service attendance among adolescents in CILS4EU across a two-year period revealed declining religiosity among Christian youth (both migrant and non-migrant), which contrasted with stability among their Muslim peers (Simsek et al. 2019). Even though a part of the Muslim youth in the four countries under study showed declining religiosity over time, an equally large part showed increasing religiosity, resulting in an aggregate trend of stability. Among Christian youth, in contrast, those showing increased religiosity were outnumbered by those who showed decline.

Altogether, the current empirical literature on religious change among immigrants shows that Muslim minorities in European societies stand out due to their higher religiosity and its greater stability over time and across generations compared to the religiosity of migrant and non-migrant Christians. A general long-term trend towards either secularisation or religious revival, however, is hard to discern from these discrepant findings rooted in different research approaches. There are only few signs of religious revival or increasing religiosity among large parts of the Muslim population, though some segments of Muslim youth do increase their religiosity over time. Findings of stability or slight—but not sweeping—decline are more common. In addition to clarifying the direction of the broad trends among the general immigrant and Muslim population in Europe, future research on religion as *explanandum* will need to move beyond its descriptive character and address the question of how individual and community differences in religious developments can be explained (i.e., why do some Muslim youth increase their religiosity during adolescents, while others decrease or stay stable?).

## Religion and immigrant integration

A second line of research considers religion as *explanans* for immigrant integration and has asked how immigrant religion relates to exposure to, participation in and orientation towards traditionally Christian but increasingly secularised European receiving societies. The overarching question in this regard is whether religion functions as a bridge towards incorporation (as has been historically the case in the US, cf. Hirschman 2004) or rather as a barrier, which has been argued to be more likely in the European context (Foner and Alba 2008). The earliest empirical studies on this topic often relied on exclusively or primarily first-generation samples, and generally showed negative correlations such that the least integrated minority members were also the most religious, in line with the barrier scenario (e.g. Phalet et al. 2008; Smits et al. 2010; Van Tubergen 2006). However, since the first generation often ‘imported’ a high level of religiosity from their (typically more religious) origin countries and bore the brunt of the costs of international migration (in terms of non-transferability of skills and networks, e.g. Friedberg 2000), this particular combination of high religiosity and lacking integration might be rather transient and rooted in the early stages of immigrant settlement. Research on the association between immigrant integration and religiosity among the second generation, or comparing first- and second-generation migrants, is better situated to examine a potentially causal role of individual differences in religious involvement for integration outcomes (cf. Voas and Fleischmann 2012).

Given the multidimensional nature of the integration concept, the body of research concerned with the association between religiosity and immigrant integration is substantial and draws on different theoretical explanations for the very distinct outcomes under study. For example, explaining differences in labour force participation requires a different theoretical and empirical toolkit than the study of national identification. Due to space limitations, I limit my review to empirical associations and do not go into the multiple explanatory mechanisms through which specific aspects of religiosity are linked to particular integration outcomes. To facilitate the review of the literature on the association between migrants’ (often: Muslims’) level of religiosity and their integration, Table 1 provides an overview of studies on empirical research articles published after 2000 that contain measures of immigrant religiosity, immigrant integration, and their association. The table is structured by integration dimension (distinguishing between structural, social and cultural); within dimensions studies are listed in chronological order of their publication date. For each study, the data source, sample, measures of religiosity and integration are listed and the main outcome is indicated in the last column, where a + sign indicates that higher levels of religiosity go together with improved integration (the bridge scenario),—indicates the opposite finding (religion as a barrier for integration) and 0 stands for non-significant associations between religiosity and immigrant integration. Since many studies investigate multiple dimensions of religiosity separately rather than using a single or composite indicator, and also often include more than one indicator of integration, there is often more than one relationship to report. The aim of Table 1 is not to provide input for a formal meta-analysis, which would be difficult to achieve given the discrepancies between measures of both religiosity and integration, but rather to create a systematic overview of the current empirical state of the art. Also note that some studies listed here stipulated a different causal order and investigated religiosity as outcome predicted by, for instance, level of education. Given the cross-sectional nature of most analyses summarised here and the goal of this part of the article, I included these studies nonetheless as they are substantively informative of the question of how levels of religiosity relate to different integration outcomes.

**Table 1:** Overview of studies on the relationship between religiosity and immigrant integration

	Author(s) (year)	Data source	Sample	Religiosity measure(s)	Integration measure(s)	Association religiosity—integration: +/0/−
Structural integration	Van Tubergen (2006)	IFIS (harmonised migrant surveys in 8 destination countries)	Nationally representative samples of immigrants (foreign-born)	Religious affiliation (yes/no), service attendance (once a week or more vs. less)	Schooling, labour force status	−: the employed and higher educated are less likely to be affiliated and to attend services frequently
	Van Tubergen (2007)^a^	SPVA 1998, 2002	First-generation immigrants from Turkey, Morocco, Suriname and the Dutch Antilles	Religious affiliation, religious attitudes (4 items), service attendance	Employed, educated in receiving country, highest level of education, language proficiency	0: affiliation with educated in receiving country, language skills −: affiliation with employed, level of education, religious attitudes and practices with language proficiency
	Phalet et al. (2008)^a^	SPVA 1998 + 2002, LAS 2005	Self-identified Muslims of Turkish and Moroccan origin	Service attendance, preference for religious school, preference for co-religious partner of one’s daughter	Educational attainment, employment status	−: higher education and working status are negatively related to all indicators of religiosity
	Diehl and Koenig (2009)	Generations and Gender Survey (Germany)	18–79 year old German speakers and Turkish citizens	Religious affiliation (Christian, Muslim, none), high religiosity (defined as 2 out 3: weekly or more frequent service attendance, agrees that religious rituals during life-course events are important and that religion is an important goal in child-rearing)	Educational attainment, labour market participation, language use	−: but only for the second generation. Higher educated, less religious, home-makers are more religious than employed
	Smits et al. (2010)	MHSM survey (Belgium), 1994–1996	Self-identified Muslim men born in Turkey or Morocco, aged 18 or older	Mosque attendance (weekly or more vs. less), participated in last Ramadan, sacrificed a sheep during Eid	Level of education, job stability	−: higher educated attend mosque less frequently and less frequently sacrifice a sheep, workers with permanent contract visit mosque less, participate in Ramadan less but sacrifice more frequently 0: no relation between education and participation in Ramadan
	Güveli and Platt (2011)^a^	SPVA (1998) & FNSEM (1993)	Self-identified Muslims (of Antillean, Moroccan, Turkish and Surinamese origin in NL, of Pakistani, Indian, Bangladeshi or East African origin in UK)	Weekly attendance at worship	Education and labour market participation, language skills	−: Netherlands 0: UK
	Fleischmann and Phalet (2012)^a^	TIES (NL, BE, DE, SE)	Self-identified Muslims who are 2nd generation Turkish and Moroccan minorities, aged 18–35	Latent variable containing religious identification (4 items), ritual and dietary practices (2 items each), and political religion (4 items)	Educational attainment, employment status	0: Belgium, Netherlands and Sweden, employment status in Germany −: educational attainment in Germany
	Lewis and Kashyap (2013b)	2008–2009 Citizenship Survey (England and Wales)	Self-identified immigrant and non-immigrant Muslims and non-Muslims	Actively practicing religion (self-report)	Educational attainment and working	0 (− among non-Muslims)
	Stichs and Müssig (2013)	Muslim Life in Germany (MLD)	25–64 year old people with migration background in one of 49 Muslim-majority countries	Religious affiliation (Muslim, Christian, none), degree of religiosity (self-report), for Muslim women: wearing a headscarf (yes/no)	Employment status, skill level of job	− for women: Muslim affiliation, religiosity and veiling associated with lower labour market participation 0: skill level of job, men’s labour market participation
	Van der Bracht et al. (2013)	ESS round 1–4	Migrants living in 26 European countries (Israel, Russia and Turkey excluded)	Religious affiliation, subjective religiosity, praying (once a week or more vs. less)	Educational attainment	−: with higher education, all aspects of religiosity decrease
	Connor and Koenig (2015)	ESS 2002–2012 in EU15+ countries	Self-identified Muslims and non-Muslims	Praying frequency (daily or more vs. less), service attendance (monthly or more vs. less) and importance of religion (1–10)	Employment (conditional on labour market participation)	−: prayer 0: importance of religion +: service attendance
	Khoudja and Fleischmann (2015)	Survey Integration Minorities	16–64 year old Dutch women without migration background and with a migration background in Turkey, Morocco, Suriname, Antilles, students, retired and permanently ill participants excluded	Mean of 3 items of religious identification	Labour market participation (yes/no), number of hours worked	−: hours worked 0: labour market participation
	Koenig et al. (2016)	SCIP wave 1 & 2	Self-identified Christian and Muslim recent immigrants from in GB (from Poland & Pakistan), Germany (Poland & Turkey), Netherlands (Poland, Bulgaria, Turkey, Morocco, Suriname, Antilles)	Service attendance monthly or more	Employment	0
	Ohlendorf et al. (2017)	CILS4EU (Germany)	Youth with immigrant background, aged ~14, comparing Muslims with migrant & non-migrants Protestants, Catholics, non-religious and other religions	Subjective importance of religion, service attendance (at least monthly vs. less frequently)	Education: type of school track, grades (Mathematics & German)	+: both religiosity measures and the three outcome variables
	Carol and Schulz (2018)	NEPS SC3 & SC4 (Germany)	Youth aged 10–13 and 14–17 with immigrant background and self-identified as Muslims, Christian or non-religious	Students’ and parents’ subjective religiosity, praying frequency and religious community engagement	Education: test performance (maths)	−: subjective religiosity 0: community engagement +: praying
	Beek and Fleischmann (2019)^a^	NELLS	1st and 2nd generation Turkish- and Moroccan-Dutch, age 15–45	Latent construct consisting of religious identification, mosque attendance, worship practices and dietary practices	Level of education, socio-economic participation, language proficiency	−: level of education 1st generation, language proficiency 0: level of education 2nd generation, socio-economic participation
	Drouhot (2021)	Trajectoires et Origines survey (2008–9) (France)	Self-identified Muslims, Christians and non-religious persons in a representative sample of immigrants (1st and 2nd generation) aged 18–60	Subjective importance of religion, dietary practices, service attendance, wearing a visible religious symbol	Family income	−: only for most and least deprived Muslims
Social integration	Van Tubergen (2007)^a^	SPVA 1998, 2002	First-generation immigrants from Turkey, Morocco, Suriname and the Dutch Antilles	Religious affiliation, religious attitudes (4 items), service attendance	Participation in majority organisation, native partner, leisure-time contacts with natives	0: affiliation with leisure-time contacts with natives −: affiliation, attitudes and participation with participation in majority organisation, affiliation with native partner
	Phalet et al. (2008)^a^	SPVA 1998 + 2002, LAS 2005	Self-identified Muslims of Turkish and Moroccan origin	Service attendance, preference for religious school, preference for co-religious partner of one’s daughter	Inter-ethnic social contacts	−: for all three indicators of religiosity
	Güveli and Platt (2011)^a^	SPVA (1998) & FNSEM (1993)	Self-identified Muslims (of Antillean, Moroccan, Turkish and Surinamese origin in NL, of Pakistani, Indian, Bangladeshi or East African origin in UK)	Weekly attendance at worship	Participation in majority-dominated association	0
	Fleischmann and Phalet (2012)^a^	TIES (NL, BE, DE, SE)	Self-identified Muslims who are 2nd generation Turkish and Moroccan minorities, aged 18–35	Latent variable containing religious identification (4 items), ritual and dietary practices (2 items each), and political religion (4 items)	Co-ethnic vs. inter-ethnic partner	0
	Maliepaard and Phalet (2012)	Survey Integration Minorities (2006), Netherlands	Turkish- and Moroccan-Dutch (1st and 2nd generation) self-identified Muslims	Religious practice (mosque visits, ramadan, halal), religious assertion (4 items), religious identification (3 items)	Contact with Dutch (friends/acquainances and neighbours)	−
	Müssig and Stichs (2012)	Muslim Life in Germany (MLD)	Self-identified Muslim and Christian first-generation migrants in Germany from 49 Muslim-majority origin countries	Frequency of service attendance, religiosity (self-report), acceptance of inter-religious marriage	Contact with German friends (once a week or more)	0: service attendance and religiosity for Muslims, acceptance of inter-religious marriage for Christians +: acceptance of inter-religious marriage for Muslims, service attendance and religiosity for Christians
	McAndrew and Voas (2014)	Ethnic Minority British Election Study (2010)	British citizens of South-Asian, African and Afro-Caribbean origin	Religious denomination, religious salience, communal and private practice	Generalised Social Trust, civic engagement and voluntary activity	0: trust +: civic engagement and volunteering
	Fleischmann et al. (2016)	NELLS	1st and 2nd generation Turkish- and Moroccan-Dutch, age 15–45	Religious service attendance	Participation in civic associations	+
	Leszczensky and Pink (2017)	Friendship & identity in school, wave 1–3	German (NRW) youth (~ 13 years) with and without immigrant background	Religious affiliation, High religiosity (daily or more frequent prayer, religious identification 3 or higher)	Friendship nominations	0 level of religiosity +: affiliation (having the same affiliation increases the likelihood of friendship nominations)
	Maliepaard and Schacht (2018)	SCIP, wave 1 & 2	Recent immigrants from Turkey to Germany and the Netherlands, and from Pakistan to England	Religious service attendance, prayer frequency (repeated measures: before migration, 2 × after migration)	Frequency of contact with host country natives	0
	Beek and Fleischmann (2019)^a^	NELLS	1st and 2nd generation Turkish- and Moroccan-Dutch, age 15–45	Latent construct consisting of religious identification, mosque attendance, worship practices and dietary practices	Superficial contact with Dutch, Dutch friends	−: Dutch friends 0: superficial contacts
	Müssig (2020)	German data from European Social Survey, round 1–7 + Polity IV	Persons with and without a migration background aged 18 or older	Religious affiliation, religious service attendance (once a month or more vs. less), self-rated religiosity (high vs. low)	Participation in last election, participation in non-electoral political activities (protest-oriented and party-based)	−: affiliation (Muslims are less politically active that non-Muslims) 0: service attendance and political participation (limited to people with migration background)
	Simsek et al. (2021)	CILS4EU, wave 1 (England, Germany, Netherlands, Sweden)	Youth (~ 15 years) with and without migration background	Frequency of service attendance, frequency of prayer, subjective importance of religion	Friendship nominations & negative ties	−: Youth who are dissimilar in religiosity and religious affiliation are less likely to be friends
Cultural integration	Verkuyten and Yildiz (2007), Study 2 & 3	Community samples of Turkish-Dutch participants (born in the Netherlands with both parents born in Turkey)	Study 2: 171 self-identified Sunni Muslims, Study 3: 191 self-identified Muslims	Religious identification (6 items) (Study 2 & 3), religious behavioural involvement (4 items), support for religious organisations (4 items) (Study 3 only)	National identification (3 items)	−: religious identification/importance, 0: behavioural involvement and political religion
	Diehl et al. (2009)	Generations and Gender Survey (Germany)	18–79 year old German speakers and Turkish citizens	Religious affiliation (Christian, Muslim, none), high religiosity (defined as 2 out 3: weekly or more frequent service attendance, agrees that religious rituals during life-course events are important and that religion is an important goal in child-rearing)	Approval of gender equality (index of 5 items)	−
	Güveli and Platt (2011)^a^	SPVA (1998) & FNSEM (1993)	Self-identified Muslims (of Antillean, Moroccan, Turkish and Surinamese origin in NL, of Pakistani, Indian, Bangladeshi or East African origin in UK)	Weekly attendance at worship	National identification	0: Netherlands +: UK
	Lewis and Kashyap (2013a)	Faith Matters (FM): British Muslims (2009)	Self-identified Muslims in areas with at least 10% Muslim inhabitants across Great Britain	Religious practices and beliefs (3 items each)	Attitudes towards gender equality, sexual minority rights, abortion, divorce, premarital sex	−
	Scheible and Fleischmann (2013)	TIES-Belgium	2nd generation Turkish and Moroccan Belgians in Antwerp and Brussels, aged 18–35, who are self-identified Muslims	Composite containing identification (4 items), practices and belief orthodoxy (2 items)	Approval of gender equality (3 items)	− (stronger among men than women)
	Becher and El-Menouar (2014)	BAMF survey (2013)	Christians and Muslims with migration background in Germany, non-migrant Germans	Subjective religiosity, religious participation, participation in rituals, importance of religion	Attitudes towards gender roles, gender equality and premarital sex, gendered division of labour within households	−
	Maxwell and Bleich (2014)	Trajectoires et Origines Suryey (France)	Self-identified Muslims	Composite measure comprising subjective importance of religion, dietary practices, service attendance, wearing a visible religious symbol	National identification (“I feel French”, dichotomised)	−
	Torrekens and Jacobs (2016)	EURISLAM	Muslims with origin from Morocco, Turkey, Pakistan, ex-Yugoslavia in CH, BEL, FR, GER, NL, UK and non-Muslim majority comparison group	Mean of religious identification, service attendance and prayer	Role of religion in society—distance from natives	−
	Eskelinen and Verkuyten (2018)	EURISLAM	Self-identified Muslims from Morocco, Turkey, Pakistan and ex-Yugoslavia in Belgium, Germany, Switzerland and UK + comparison sample of Christian natives	Religious identification (2 items), and religious practices (prayer, attendance, hair cover, no alcohol, celebrating holy days, wearing religious symbols, following dietary restrictions)	Support for democracy (2 items) and liberal sexual mores (4 items)	−
	Fleischmann and Phalet (2018)	CILS4EU + LeuvenCILS	Youth (~ 14) with and without migration background in England, Germany, Netherlands, Sweden and Flanders	Religious affiliation (Christian, Muslim, other, non-religious), frequency of service attendance, frequency of prayer, subjective importance of religion	National identification	−
	Kogan and Weißmann (2019)	CILS4EU, wave 1 & 3 (England, Germany, Netherlands, Sweden)	1st, 2nd, and 3rd or higher immigrant generation youth aged ~ 15	Religious denomination, subjective importance of religion	Acceptance of premarital cohabitation	−
	Beek and Fleischmann (2019)^a^	NELLS	1st and 2nd generation Turkish- and Moroccan-Dutch, age 15–45	Latent construct consisting of religious identification, mosque attendance, worship practices and dietary practices	Gender egalitarianism, sexual liberalism, national identification	−: sexual liberalism 0: gender egalitarianism, national identification

Studies marked with ^a^ examine more than one dimension of immigrant integration and are therefore listed repeatedly. The organisation of outcomes into structural, social and cultural dimensions of immigrant integration is imperfect. Some indicators could be argued to belong to multiple dimensions (e.g. language proficiency, currently listed under structural) and some outcomes could be considered a separate dimension (e.g. political participation) but were not listed separately due to a lack of relevant studies

The first dimension considered in Table 1 is structural integration, referring to educational achievement and attainment, labour market positions, family income and language skills. A number of studies document negative associations with immigrant religiosity such that the most religious (Muslim) immigrants have the lowest levels of education and labour market participation (e.g. Van der Bracht et al. 2013; Van Tubergen 2007). In France, negative relations between religiosity and family income were also found, but these were restricted to the most and least deprived subgroups within the Muslim population (Drouhot 2021). A comparative study of second-generation Turkish Muslims found negative associations of religiosity with educational attainment in Berlin—but no significant associations in Amsterdam, Brussels and Stockholm (Fleischmann and Phalet 2012). A decoupling of religiosity and educational attainment was likewise found in studies among Muslims in the UK (Lewis and Kashyap 2013b) and immigrant youth belonging to multiple denominations in Germany (Ohlendorf et al. 2017). The German NEPS-study even shows positive associations between adolescents’ frequency of prayer and scores on a math test, but simultaneously reveals a negative relation of subjective religiosity and a non-significant one of community engagement with test scores (Carol and Schulz 2018).

With regard to employment, there are also differential outcomes between different aspects of religiosity as well as between genders. More frequent prayer was associated with lower, but more frequent mosque attendance with higher chances of being employed among Muslims in Western Europe (Connor and Koenig 2015). Among Muslims in Germany and ethnic minorities in the Netherlands, greater religiosity (and, in Germany, also wearing a headscarf) were negatively related to women’s labour market participation (Becher and El-Menouar 2014; Khoudja and Fleischmann 2015), but unrelated to that of men (Stichs and Müssig 2013). Longitudinal research among recent immigrants further points towards decoupling, as no significant relation between the religiosity of Polish Christians and Turkish Muslims and their chances of being employed was found across three destination countries (Koenig et al. 2016). For the domain of structural integration, thus, there are conflicting and partly gender-specific findings including negative, positive and non-significant relations of (Muslim) immigrants’ religiosity with their educational and labour market outcomes and language skills.

This lack of clear and strong relations may be attributable to the closer links between religion and integration in the socio-cultural domain, and much research has been conducted in this field as well. When it comes to the contacts with out-group members that immigrants maintain, network analyses show that Muslims are most segregated from Christian and non-religious peers in diverse school classes (Leszczensky and Pink 2017; Simsek et al. 2021). Some survey studies show that more religious Muslims have less contacts with natives (Maliepaard and Phalet 2012; Phalet et al. 2008; Van Tubergen 2007), but others find no significant associations between Muslim immigrants’ religiosity and their interethnic contacts (Fleischmann and Phalet 2012; Güveli and Platt 2011; Müssig and Stichs 2012; Maliepaard and Schacht 2018). Regarding participation in voluntary or civic organisations, another common operationalisation of social integration, positive correlations with religiosity were found among Turkish and Moroccan Muslims in the Netherlands (Fleischmann et al. 2016), and among 1.5 and 2nd generation ethnic minorities in the UK (McAndrew and Voas 2014), but there are also competing findings indicating decoupling (Güveli and Platt 2011) or negative correlations (Van Tubergen 2007). Examining electoral and non-electoral forms of political participation among people with a migration background in Germany, Müssig (2020) finds no relation between the frequency of service attendance and political participation among people with a migration background, which contrasts with a positive association among non-migrant Germans. In sum, for social integration, both in terms of contacts, partner choice and participation in organisations and politics, we again observe mixed findings including positive, negative and non-significant associations with (Muslim) immigrants’ religiosity.

In contrast, the relation with religiosity is consistently negative with regard to specific social attitudes that demarcate the boundary between conservative and progressive factions of the population. Thus, more religious (Muslim) immigrants hold more conservative values regarding issues of sexual liberalism, such as the acceptance of premarital sexual relations, homosexuality and abortion (Becher and El-Menouar 2014; Beek and Fleischmann 2019; Eskelinen and Verkuyten 2018; Kogan and Weißmann 2019). Regarding gender equality, findings are more mixed and include negative (Becher and El-Menouar 2014; Diehl et al. 2009; Lewis and Kashyap 2013a; Scheible and Fleischmann 2013) as well as non-significant associations (Beek and Fleischmann 2019). Importantly, many of the studies on social attitudes compare the association of religiosity and attitudes between migrants and majority members and find similar associations of religiosity with conservatism among (largely Christian) natives (e.g. Diehl et al. 2009; Lewis and Kashyap 2013a). Thus, while immigrants in general and Muslims in particular seem to be more conservative on average than non-migrants, this conservatism seems to result from a composition effect and their religiously-inspired conservatism does not distinguish them from similarly conservative and religious non-migrants.

In addition to social attitudes, many studies looked at national identification, a conceptually different but societally relevant outcome in the domain of cultural integration. National identification has been found to be negatively related to religiosity among adult samples of Muslims (e.g. Maxwell and Bleich 2014; Verkuyten and Yildiz 2007), and Muslim and non-Muslim youth across European societies (Fleischmann and Phalet 2018). Again, contrasting findings are available that show non-significant (Beek and Fleischmann 2019; Güveli and Platt 2011; Dutch results) or even positive (Güveli and Platt 2011; UK results) associations between Muslims’ religiosity and their level of national identification. Also with regard to national identification, then, the findings regarding the role of religiosity can only be characterised as ‘mixed’.

The upshot of 15 years of large-scale empirical research on religious changes among immigrants in general, and Muslims in particular, is thus rather puzzling. On the one hand, there is strong evidence for the continued importance of religion among immigrants in general and Muslims in particular, and for comparatively higher levels of religiosity and stability among Muslims compared to non-Muslims. On the other hand, it is still unclear how and why this matters for specific aspects of their integration into European societies. This is a pressing problem for migration scholars as religion continues to be important from the migrant perspective and in public debates where it acts as most important fault line to discuss cultural differences (Brubaker 2015). In the final part of this contribution, I therefore propose a research agenda to address this pressing question.

## Extending existing research on religion and migration: a research agenda

My proposal for a future research agenda is twofold and contains a conceptual and an empirical element. Empirically, a welcome extension would be to broaden the scope of the religious minorities under study beyond the Muslim case. Previous studies on religion and immigrant integration in Europe have—mostly for practical reasons of data availability—focused on (predominantly Sunni) Muslims. The focus on Muslims is not only pragmatic but also societally relevant as the divide between Muslims and non-Muslims is the most prominent fault line in the polarised public opinion landscape regarding religion and immigrant integration (e.g. Brubaker 2015; Foner and Alba 2008). This singular focus, however, comes at the cost of understanding the role of religion in general—rather than (Sunni) Islam in particular—for immigrant integration, and it risks glossing over important internal distinctions among a global religious community characterised by a high level of internal diversity. Future research should therefore include other religious minorities, such as Hindus, Orthodox Christians or non-Sunni Muslims, and more systematically compare Sunni Muslims with immigrants and non-migrants of other religious affiliations in terms of their levels of religiosity, trends in religiosity and its relationship with multiple dimensions of immigrant integration.

The second innovation I propose is of conceptual nature, where I suggest to enhance the large-scale quantitative study of immigrant religion with more than counting practice frequencies and importance of religion, and include measures of religious orientations, reasoning and meaning-making. This proposal follows the classic account of William James (1902) that rather than what individuals believe, the way in which they hold their beliefs is important in understanding the social function of religion. Previous research that documented how the association between religiosity and integration outcomes changes across immigrant generations (e.g. Beek and Fleischmann 2019; Voas and Fleischmann 2012) already suggested that the relevance of religiosity for integration changes from the first to the second generation. However, changing strengths or directions of associations between religiosity and e.g. social attitudes are a rather indirect test of changed meanings of religion, and they do not provide insights into how, why and in which aspects religion changes over time. Qualitative research on immigrant youth’ religion already showed that they take different approaches towards ‘being a Muslim’ (e.g. De Koning 2008; Peek 2005; Vertovec and Rogers 1998). To some extent, such differences can be replicated with quantitative methods using person-based analytical techniques such as (latent) profile or cluster analysis to construct typologies of Muslims (e.g. Huijnk 2018; Maliepaard and Gijsberts 2012; Phalet et al. 2012). Yet with the measures currently available, such person-based analyses are still limited to what people do and how often in the realm of religion, and how much importance they attach to it, but they do not shed light on the way in which people reason about religion.

Extending the study of immigrant religion with a focus on religious orientations and the meanings that immigrants derive from religion could potentially resolve some of the conflicting findings identified in the previous section. The research summarised in Table 1 has focused on measures of the subjective importance of religion and the frequency of participation in specific religious practices, most often referring to service attendance and prayer, but sometimes also including practices such as fasting, sacrificing or reading scripture. Consequently, previous studies have missed out on changes in the meaning of religiosity that could occur over time and across migrant generations. Consider the example of a father and son who both visit the mosque every week for Friday prayer. Conventional research would interpret this as a sign of intergenerational stability and lack of change in religiosity, based on the identical frequency of their service attendance. However, if the father visits a liberal and the son a radical Salafi mosque (or the other way around), this constitutes a relevant religious change across generations. This meaningful change would, however, not be evident in studies using existing research instruments as they do not include the meanings that individuals derive from their religion and how they reason about it. To comprehensively understand religious change, which is broader than simply religious decline (Dobbelaere 2002), in the context of migration, I argue that we need to better understand individuals’ reasoning about religion instead of focusing exclusively on their levels of religiosity.

According to Wulff’s (1997) seminal overview of the psychology of religion, religiosity is only one of two core dimensions in individuals’ orientations towards religion. The second is religious cognition, which ranges from literal to symbolic. Literalists insist that there is only one correct answer to religious questions (‘one truth’), whereas symbolists emphasise the need to (re-)interpret religious messages and acknowledge the value of multiple worldviews. Individuals with more literal religious cognitions tend to hold more stereotypical worldviews and avoid questioning their convictions, whereas those with more symbolic cognitions are more open to challenging their worldview and adapting their attitudes and behaviours based on new information (Batson and Raynor-Prince 1983; Hunsberger et al. 1996). A symbolic religious cognition thus reflects “a tendency for people […] to think complexly both about religion and about people and diversity” (Hunsberger and Jackson 2005: 816). This renders religious cognition of paramount importance for immigrant integration, which essentially requires individuals from different (religious) groups to come to terms with the diversity of their surroundings. However, unlike religiosity, religious cognition has not yet been systematically investigated among immigrants and their offspring, neither has it been examined in relation to immigrant integration.

Outside migration studies, the notion that religious attitudes or religious cognition are relevant beyond individual differences in religious involvement is already more established. In their overview of research using Hutsebaut’s (1996) Post-Critical Belief Scale (PCBS), which is modelled on Wulff’s approach, Duriez et al. (2007) found the dimension of literal vs. symbolic religious cognition to be more predictive of individuals’ attitudes (e.g. prejudice), values (e.g. universalism) and behaviour (e.g. party choice) than religiosity. However, studies with this instrument have been limited to non-migrants with Christian backgrounds (Krysinska et al. 2014), also due to the fact that some of the PCBS-items explicitly refer to Christian contents (e.g. the Bible, priests, Mary, Jesus). Using different measures of religious attitudes, Pickel et al. (2020) found that dogmatic and exclusivist approaches to religion are more relevant for understanding prejudice than religious affiliation and religiosity among majority samples of the German and Swiss population.

Dogmatism, exclusivism and literalism resemble the concept of religious fundamentalism, which has already been studied in the context of migration, where it was shown that fundamentalist attitudes are rather widespread among Sunni Muslims in Europe (Koopmans 2015). However, in contrast to the two-dimensional approach proposed here, religious fundamentalism always presupposes a certain level of religiosity (e.g. Altemeyer and Hunsberger 2004; Moaddel and Karabenick 2021). Therefore, existing measures of religious fundamentalism are unsuited to explain differences in integration among immigrants who score low on religiosity—but who can still reason in different ways about truth and meaning, with potentially important repercussions for their integration into diverse societies. Moreover, research on immigrants’ fundamentalism has been limited to the explanation of out-group hostility and has not considered other dimensions of immigrant integration. Yet a lack of prejudice is only one component of successful integration, and it is still unclear how differences in religious cognition relate to equal access to resources, interethnic social ties and convergence in attitudes and identification as other key indicators of immigrant integration (Alba and Nee 2003; Gordon 1964). Based on research among non-migrants and previous work on religious fundamentalism, I expect that religious orientations such as literal vs. symbolic religious cognition will be more strongly and consistently related to immigrant integration than religiosity. More specifically, symbolic, inclusive and non-dogmatic approaches to religion should be more positively related to e.g. interethnic contacts and liberal social attitudes than literal, exclusive and dogmatic approaches.

The conceptual extension proposed here will bring the field closer to examining religious contents, rather than focussing only on levels of religiosity. A useful analogy to understand the added value of this innovation is the work on national identity contents, where the distinction between ethnic, civic and cultural criteria for national belonging helped to understand why some but not all individuals who are more strongly nationally identified hold more negative attitudes towards immigrants (e.g. Reijerse et al. 2013). Similarly, those who subscribe to more literal or exclusivist interpretations of their religion might be more oriented towards their religious in-group and less willing to engage in contact with out-group members (e.g. Kanol 2021), hold more conservative attitudes e.g. with regard to sexual minority rights (e.g. Pickel et al. 2020), which might translate into more strongly gendered patterns of labour market participation (e.g. Khoudja and Fleischmann 2015). More generally speaking, it is plausible that, if defined in an exclusivist, literalist or fundamentalist way, religion is a barrier for immigrant integration, particularly in domains that require cross-religious contacts or concern attitudes that are considered central to one’s religious teachings. If defined in pluralistic, inclusive or symbolic ways, religion instead is more likely to be decoupled from immigrant integration or even has the potential to be positively related, forming a bridge towards inter-ethnic contacts, participation in associations and identification with a shared national identity.

## Conclusion

Since the start of the current millennium, migration research on immigrant religion has taken a turn from an initial focus on institutions and legal accommodation towards the study of religiosity among the ‘average immigrant’ or ‘average Muslim’, facilitated by an increasing number of indicators of religiosity in large-scale surveys that include sufficient samples of immigrants and Muslims. This body of research has shown that Muslim minorities in European societies are distinct from non-Muslims (both immigrant and non-migrant) in their higher levels of religiosity and greater stability of religious identification, practices and attitudes. Due to a limited focus on religious importance and the frequency of religious practices and a neglect of individual differences in religious orientations, however, scholarship in this field has been unable so far to formulate a conclusive answer to the question of how and why religion matters for the integration of immigrants into European societies. The strong focus on Muslims as largest religious minority in Europe has further raised the question whether the associations found so far are generic to religion or particular to (Sunni) Islam. The research agenda for the future should therefore broaden the empirical scope to include other religious minorities than (Sunni) Muslims and more systematically compare Muslim immigrants to those with other religious affiliations. A second needed innovation is a stronger focus on the meaning of religion that can be achieved by a different conceptual approach to the phenomenon that focuses more on the way in which immigrants relate to their religion and derive meaning from it.

## Footnotes

1 Western, Educated, Industrialised, Rich and Democratic (cf. Henrich and Norenzayan 2010).
